# Who Cares for Communities? Conceptualizing Non-Profit Work as Social Reproduction Through the Case of Food Banks

**DOI:** 10.1177/08861099251340703

**Published:** 2025-05-13

**Authors:** Meghan Mendelin, Rebecca Jane Hall

**Affiliations:** Global Development Studies, 4257 Queen's University, Kingston, Canada

**Keywords:** social reproduction, care work, non-profit organizations, neoliberalism, food banks

## Abstract

This article conceptualizes non-profit labour as a form of social reproduction, a feminist political economy category that refers to the labour involved in the making of life and tending to it. In the contemporary context of neoliberal austerity in Canada, non-profit organizations have increasingly stepped in to fill widening gaps in essential social provisioning. The predominantly female non-profit workforce within these organizations, parallel to feminized household labour, faces systemic undervaluation and invisibilization, reflected in low wages, chronic underfunding, and austerity policies that work to destabilize the sector as a whole. Via the case study of food banks, we seek to make visible these vital, life-sustaining and community reproducing labours, drawing attention to their political agency and social power. While we write from within critiques of food banks as a symptom of neoliberal retrenchment, we resist conflating the labour and relationships within these organizations with the critiques of the political economic conditions in which they operate. Instead, we argue that the labours of social reproduction that occur within food banks hold political potential that is at odds with the (ostensibly) depoliticized approach to charity that is favoured in neoliberal modes of social provisioning. Providing food for communities, like other social reproduction activities, is not merely a response to gaps in formal government or market provisioning, but is agentic life-making labour driven by dictates beyond capital accumulation.

Social reproduction refers to the multitude of labours involved in the making and sustaining of life. These labours are essential to the functioning of the global capitalist political economy (Ferguson & McNally in Vogel, 2013). Despite its fundamental importance, social reproduction is systematically marginalized and devalued under capitalism, occupying what Vosko (2016) describes as a liminal space – simultaneously central to the economy and rendered invisible within it. Labour associated with social reproduction – nurturing, teaching, cleaning, feeding, and caring – tends to be both obscured and exploited (Rai, 2024). Historically, social reproduction has often been equated with household domestic labour (Bezanson & Luxton, 2006). However, a growing body of literature makes visible the multiple scales, sites and forms through which life-sustaining work occurs. This involves thinking through social reproduction beyond the proverbial white picket fence to include the labours that sustain communities, often occurring in formal and informal organizations that reproduce life on a daily and intergenerational basis.

Feminists have long sought to reveal these labours, their exploitation, and their political power. For example, Nina Banks (2020) examines how Black women's unpaid labour in the United States – caring for their communities and organizing to fight systems of oppression – must be recognized *as work* (emphasis added). Similarly, Sophia Schmid (2020), writing on Germany, argues that refugee support work, a voluntary form of labour primarily performed by women, constitutes a form of unpaid care work that holds transformative power and can counteract xenophobic stereotypes. Such studies illustrate how social reproduction operates not only as an exploited form of labour, but also as a site of political struggle and collective resistance. Thus, while the demands of capital shape our lifemaking labours, the imperatives driving social reproduction – both intimate and political – extend far beyond capital, rooted instead in the ongoing needs of communities and social life itself.

In this piece, we engage the analytic of social reproduction to examine the community labours performed in non-profit organizations. We situate our analysis in the context of large-scale state withdrawal from the provision of public goods and services under the dominant political-economic ideology of neoliberalism (Harvey, 2005). Our geographic focus is Canada, though the politics of austerity are shared by similar highly industrialized post-welfare state countries. By conceptualizing the work performed by non-profit organizations as a form of social reproduction, we highlight the essential caring work that the female-majority workforce in this sector performs. Like caring work that is performed in the household, caring for communities through non-profit, charitable and social work in community-based non-profits is overwhelmingly carried out by women, who make up 77% of this workforce in Canada (Jensen, 2022).^1^ These non-profits are engaged in a range of social provisioning activities. Recognizing that these activities operate through multiple, and sometimes conflicting ideologies (from mutual aid to charity, religious and not), numerous non-profits’ programming is centred around the provision of food, clothing, shelter, basic safety, health care, child care, or elder care. Social service non-profits play a crucial role in supporting society's most marginalized populations, such as visible minorities, refugees, and houseless people.

With a focus on community-based organizations, and via the case study of food banks, we argue that non-profit organizations play an indispensable role in caring for and reproducing our communities. This role is carried out, and made increasingly necessary, through and against the constraints of neoliberal austerity. While numerous studies have commented on the growing role of non-profit organizations in meeting the needs of society in the context of neoliberal state withdrawal (e.g., Baines et al., 2020; Bernal & Grewal, 2014; Gallagher et al., 2024; Strong, 2020), few have conceptualized non-profit work explicitly as social reproduction (Baines et al., 2020; Chakraborty, 2021). In theorizing this labour as social reproduction, we aim to make a number of interventions: first, in situating this labour as necessary for the reproduction of communities, we aim to politicize the labours of non-profits that are often made benign through feminized demarcations of “service” or “charity”. Second, by contextualizing this labour in the neoliberal political economy of austerity, we aim to make visible both the shifting and unevenly distributed gaps in state provisioning, and the community labours – and labourers – that have mobilized to both fill these gaps and, at times, reshape them. Non-profit work is often obscured and devalued by its relationship to (by definition, unpaid) volunteer labour or charity; however, by focusing on non-profit labour, which can be both paid and unpaid, our third aim is to complicate the paid/unpaid and formal/informal (Mezzadri, 2021) divide in labour studies, locating our subject in relation to lifemaking, rather than the accumulation of capital.

The first part of the paper lays the theoretical groundwork for conceptualizing non-profit work as a form of social reproduction. The second part of the paper offers an analysis of food banking from a social reproduction perspective. Food banks have been rightly criticized as a symptom of neoliberal retrenchment and, in many cases, as a depoliticized model of social provisioning. While our own analysis aligns with this critique, we seek to contribute a focus on the feminized labours (both paid and unpaid) upon which these institutions rely, suggesting that the actual caring labours at work in these organizations are erroneously robbed of their political agency and social power when they are conflated with the structures in which they work. We aim to provide an opening to pay close attention to these labours as sites of care and connection that operate in spite of and in contradiction to, the (ostensible) depoliticization of social provisioning.

## Conceptual Framework: Social Reproduction

The concept of social reproduction encompasses the multitude of activities involved in the making and sustaining of life. It entails the provision of food, clothing, shelter, basic safety, health care, biological reproduction, caring for dependents, all forms of household labour, the unpaid production of goods and services in the home, the development and transmission of knowledge, and the reproduction of culture and ideology (Bezanson & Luxton, 2006; Elias & Rai, 2019; Winders & Smith, 2019). While heteropatriarchal, European constructions of care often locate this labour in private home spaces, social reproduction scholars have demonstrated the multiscalar (Hall, 2022; Strauss, 2013) and multi-sited (Mezzadri, 2021; Rai, 2024) nature of social reproduction, as well as the tight links between household, or intimate, labours and the global political economy (Elias & Rai, 2019).^2^ Indeed, it is often colonial and racist forces that *make* social reproduction nuclear (in appearance, at least), at the expense of place-based and culturally-grounded inter-household, kin-based and community forms of reproduction (Hall, 2022; Nahanni, 1992). The multi-scalar and political nature of social reproduction is especially evident when examined from the perspective of racially and economically marginalized communities. As Patricia Hill Collins (2000) writes, for Black communities in the United States, the labours that reproduce them are political labours of resistance. These labours operate both within and beyond the political economic imperatives of capitalism, responding to dictates of community care (Taylor, 2018) and accountability.

Central to the social reproduction lens is the assertion that the production of goods and services cannot be extricated from the unpaid labours that are performed to sustain and reproduce lives. Challenging gender-blind Marxist political economic theory, social reproduction theorists assert that, in addition to workers and capitalists who own the means of production, there are people, mainly women, who produce and maintain the labour power that operates in the productive sphere (Bakker, 2007; Bhattacharya, 2017). Early European social reproduction theorists advanced the idea that capitalist production is predicated on the reproductive household labours performed by women, thereby rejecting the capitalist logic that considers wage labour an unproduced resource and only recognizes labour done for the market as a legitimate form of work (Federici, 1975; Mies, 1986; Vogel, 2013). Moreover, these scholars and advocates illustrated how the labours of social reproduction are structurally devalued and invisibilized despite their role in allowing capitalism to function and be sustained (Elias & Rai, 2019).

Black feminisms, Indigenous feminisms, and more recent scholarship on social reproduction reach beyond the household to examine sites at which production and reproduction converge and merge; for example, noting the commercialization and commodification of certain social reproductive functions in the form of take-out food, commercial laundromats, and paid domestic workers (Winders & Smith, 2019). As Claudia Jones wrote, too, for some women – especially Black women and working class women – domestic labour is also wage labour (Boyce Davies, 2007). Longstanding spaces of productive/reproductive convergence operate alongside newer transformations wrought by neoliberalism, such as the erosion of the social wage and large-scale state withdrawal from the provision of public goods and social services (Fraser, 2016). In Canada, for example, the neoliberal shift beginning in the 1980s was partially characterized by a drastic reduction in funds allocated for social services, particularly through the elimination of the Canada Assistance Plan (CAP) in 1995, and stricter eligibility criteria for social security programmes (Carson, 2014).

With the rise of neoliberalism, scholars identify a privatization of social reproduction, as the responsibility for care work is ‘downloaded’ from the state to families and communities (Bakker, 2007). This neoliberal reorganization should not be read as an even process of state retrenchment; as Sunera Thobani (2007) notes, state social spending has always operated through racial, class and colonial hierarchies, targeting some for provisioning and others for surveillance and punishment. In Canada, Indigenous households have long been a site of state surveillance and punishment, social welfarist activities that have not dissipated in the neoliberal era (Blackstock, 2007). Much contemporary work in feminist political economy is thus concerned with how this iteration of capitalism reinforces and/or exacerbates existing inequalities and spurs the crises to which capitalism is prone (Bakker, 2007; Dowling, 2021; Fraser, 2016). The social interventions that these crises necessitate, we argue, is in part being shouldered by community-based non-profit organizations and social service organizations who provide essential goods and services at a low or no cost, be it in the form of clothing, shelter, child care, or, as the focus of this paper, food. We consider the ways in which non-profit workers contribute to the social reproduction of their communities and – under and through the constraints of neoliberal austerity – work to reshape those communities.

## A Social Reproduction Approach to Non-Profit Labour: From Wages for Housework to (Better) Wages for Non-Profit Work

Under the patriarchal and racial logics of our capitalist conjuncture, social reproduction – whether it be paid or unpaid, in the formal or informal sector, and at all scales – is consistently undervalued labour, as only labour produced for the market is considered productive and, thus, “legitimate ‘work’” (Bhattacharya, 2017, p. 2). The radical feminist political movement ‘wages for housework’ sought to challenge this disregard for the socially and economically necessary labours performed by women by demanding a pay for housework and childcare (Federici, 1975). Like these labours within the home, the undervaluation of care work is articulated within non-profit organizations through the poor remuneration workers receive in this sector. In numerous studies, Donna Baines highlights the consistently low and stagnant wages of the predominantly female non-profit workforce. For example, a study on social service work in South Africa shows that workers were being paid only a ‘stipend’ amounting to roughly 60% of the minimum wage, leaving them struggling to provide food and basic necessities for their families (Baines et al., 2020). Another study on non-profit elderly care organizations in Australia documents the low wages that the female-majority workforce has to contend with in this profession (Baines et al., 2022). Certainly, the gendered-ness of a sector characterized by chronically poor wages cannot be overlooked. As Baines et al. (2020) write, “Like in the home and community, the majority female non-profit care workforce is assumed to have an endless capacity to care, regardless of pay or working conditions” (p. 453).

We consider the poor remuneration in the non-profit sector a reproduction of what Maria Mies (1986) calls ‘housewifeization’, in which any labour performed by women is considered synonymous to housework, and therefore unproductive, unskilled and unworthy of (adequate) pay. Federici (2004) links this devaluation to capitalist accumulation, which relies on an ‘accumulation of differences’ that enables ‘hyper-exploitation’ based on differences of race, sexuality, gender, class, age, citizenship and ability. Since Mies's and Federici's foundational work, scholars have tracked the ways in which the devaluation and naturalization of women's social reproductive labour in the domestic sphere intersects with the devaluation of feminized caring labour and labourers, such as daycare workers (Perrier, 2022). This devaluation articulates through racial and spatial hierarchies that mark some women's caring labour as both *more* natural and *less* valuable than others (Tungohan, 2023, emphasis added). The transnational regime of migrant care work is a potent example of this, wherein women of the Global South provide necessary caring labour (childcare, as well as elder care and personal support work) to the Global North through exploitative regimes that mobilize racial and gendered hierarchies alongside citizenship and place (see Tungohan, 2023).

This long tradition of undervaluing and invisibilizing the feminized labours of social reproduction extends to the non-profit space, as well as the social work sector, in which work resembles the caring labours performed in the household and the workers are often women. Like the imagined private sphere of the home, care work organizations operate on the naturalized, gendered expectation of the unending emotional labour expected of all women, regardless of wages, working conditions or other responsibilities. As Mies (1986) asserts, these gendered and racialized processes of devaluing this labour are furthered by the liminality of much caring labour in the capitalist economy: that is, that it can be both paid and not, formal and informal. Much community labour is unpaid, and, like paid non-profit labour, volunteer labour operates through a range of contradictions: while, in some iterations, a depoliticized replacement for formerly state-services, volunteering can also be a marker of social and political engagement (e.g., Schmid, 2020 on refugee volunteer work). In either iteration, it is essential labour for the wellbeing of communities. While it is beyond our scope to delve further into these contradictions, we raise volunteer labour because of its capacity to both replace paid non-profit jobs and to undermine their value (“If someone will do this for free, why are we paying you so much for it?”), two tendencies that work in concert.

## Non-Profit Labour Under and Against Neoliberalism

Restructuring of public funding under neoliberalism has intensified the undervaluation of social reproduction, especially at the community scale. During Canada's Keynesian era (1940s-1970s), economic policies prioritized state involvement in the market and social spending on government programmes (Gill, 2021; Osberg, 2021).^3^ This period saw increased public funding for social services, shaping non-profit organizations as extensions of state welfare rather than as substitutes for government support (Shields, 2014). Non-profits were supported by the state through long-term funding relationships that covered their core operations as well as their overhead costs (Shields, 2014). Since the 1990s, government funding for Canadian non-profits has drastically declined and core funding has been replaced with short-term project-based funding that narrowly prescribes how funding can be spent (Gallagher et al., 2024; Shields, 2014). Moreover, fewer funding sources are available for non-profits in general, fuelling immense competition among organizations (Baines & Cunningham, 2020; Shields, 2014; Tulli-Shah et al., 2024).

These shifts have left non-profits chronically underfunded, despite their growing responsibility in the absence of state support for social assistance. For example, Richmond and Dokis's (2023) study on an Indigenous food bank programme in London, Ontario, reveals the financial strain caused by the restrictions and limitations of project-based funding. Operated by two staff members from the Southwest Ontario Aboriginal Health Access Centre (SOAHAC), the food bank plays an essential role in providing culturally significant foods to Indigenous people in the area, whose food insecurity reached crisis levels during and after the pandemic. However, because the project funding for the food bank does not cover human resources, SOAHAC staff must take on unpaid workloads to keep the programme running, working during lunchbreaks, evenings, and weekends, placing immense pressure on both the workers and the organization. This funding arrangement reflects the capitalist logic that naturalizes social reproductive labour into nonexistence (Bhattacharya, 2017) – by structuring grants in a way that elides the need to pay those performing the work, assuming that care and community support will simply happen, without recognition or compensation. In Baines and Cunningham’s (2020) words, there exists a gendered expectation that the female-majority workforce “will work well beyond their paid hours in ways that are not dissimilar to expectations of boundless, unpaid female care work in the home and community” (Baines & Cunningham, 2020, p. 437). Underfunding (as a result of undervaluing) can also have health and safety implications. Kosny and MacEachen (2010), in their study on non-profit social service organizations in Toronto, argue that the occupational health and safety policies in non-profits are seriously lacking due to gendered assumptions in funding arrangements around care work as naturally low-risk and safe. This is indicative of the invisibility of the actual labours that are carried out within non-profits, many of which do involve significant physical and emotional risks, such as exposure to workplace violence, physical strain from caregiving tasks, burnout, vicarious trauma, and chronic emotional exhaustion. Such harms are compounded by precarious working conditions and minimal institutional supports, making the cost of care labour both immediate and enduring for workers themselves.

Despite the constraints through which non-profit workers have to operate, some scholars argue that non-profits constitute a powerful form of gendered resistance to the social uncaring that is rampant in the neoliberal context. In Baines et al.'s (2020) case study, a dispute with the funder left workers without pay for months, yet they continued working because they felt they could not abandon the people they served. Similarly, Kosny and MacEachen (2010) found that despite the emotional strain of working in an underfunded environment, workers saw curtailing their services as unimaginable because of their clients’ needs. Such dedication to clientele contradicts the capitalist framework that epitomizes profit and wage-based labour relations: while this dedication is a real and potential site of hyper-exploitation, it also exemplifies commitments to communal care as a ‘site of governance’ (Taylor, 2018, p. 79) unto itself. Moreover, non-profit workers transcend traditional dichotomies of labour by engaging in paid and unpaid forms of care work. This echoes the long history of Black and other racialized women's negotiation of the private-public divide, where care work extends beyond the household into community spaces (Banks, 2020; Hill Collins, 2000; Naples, 1992). As Naples (1992) describes through the concept of ‘activist-mothering’, Black and Latina women have historically engaged in community care and advocacy that not only supports collective survival but actively combats systems of oppression. Similarly, contemporary non-profit workers – particularly women – find themselves performing essential but underrecognized labour that sustains marginalized populations while also challenging the ideologies that necessitate them.

The underfunding of the non-profit sector is a striking marker of the political and social devaluation of non-profit work, the misrecognition of the complexity and importance of the work, and an underestimation of this sector's indispensability, particularly given its distinctive role in the neoliberal period. Simultaneously, neoliberal funding policies paradoxically work to destabilize the sector's existence. But non-profits also serve as key sites of resistance to the neoliberal capitalist ideology that marginalizes their labour, makes them benign through feminized demarcations of ‘charity’ or ‘volunteerism’, and dismisses their economic significance. This framing contributes to the (perceived) depoliticization of non-profit labour. However, as we demonstrate via the case of food banks below, the social reproduction activities of non-profits are *inherently* political, as they operate both within and against the gendered and racialized constraints of the neoliberal political economy, privileging life over accumulation through everyday labours and relations.

## Food and Care in Austere Times: Banking on Food Banks

Food insecurity, that is, a lack of access to sufficient nutritious food due to financial constraints, has become a growing problem in many high-income countries over the last 40 years (Long et al., 2020; Strong, 2020). Food aid and food charity have become the predominant response to this issue in the Global North, particularly in the form of food banks (Cloke et al., 2017; Long et al., 2020). Food banks vary in organizational structure across geographic contexts, but can broadly be defined as charitable organizations that receive food donations to be distributed to food insecure individuals and households (Tarasuk et al., 2020). Other non-profit food provisioning organizations such as free meal providers and food justice groups have also come to play a significant role in addressing food insecurity (Spring et al., 2022). Rising food insecurity and the increase of food banks and food organizations is widely attributed to neoliberal policymaking and welfare retrenchment (Lambie-Mumford, 2019; Regnier-Davies et al., 2023; Tarasuk et al., 2020). In Canada, food banks proliferated in the 1980s and 1990s alongside social policy reforms that severely weakened the social safety net, such as the elimination of the CAP in 1995 (Tarasuk et al., 2020). While initially intended to provide a temporary solution to food insecurity, food banks in Canada have become a permanent fixture in the landscape of social support, now deeply embedded in the fabric of our neoliberalized welfare system (Azadian et al., 2023; Regnier-Davies et al., 2023; Tarasuk et al., 2020).

Food insecurity in Canada is steadily on the rise, with the percentage of people living in food-insecure households in the ten provinces increasing from 16.8% in 2019 to 22.9% in 2023 (University of Toronto, 2024). The highest percentage of food-insecure households was found among Black people at 40.4% and Indigenous peoples at 36.8%, though the latter statistic is likely inaccurate (and much higher) given an underrepresentation of people living on First Nation reserves and remote Northern communities in government data, as well as lack of data on the territories (University of Toronto, 2024). Food bank usage has drastically increased over the last few years. In March 2023, there were almost 2 million visits to food banks across Canada, representing a 32% increase from March 2022, and a 78.5% increase from March 2019 (Food Banks Canada, 2024). The impact of the COVID-19 pandemic is evident in these numbers, yet even in the post-pandemic context, the demand for charitable food programmes continues to rise (Regnier-Davies et al., 2023). Notably, in March 2024, 61% of those who had recently accessed a food charity programme were doing so for the first time (Ovid, 2024).

Although food banks and other non-profit food organizations play a crucial role in addressing food insecurity, they remain dependent on precarious and unsustainable funding arrangements. The government's role in funding for food banks can be described as mostly facilitative, supporting them indirectly by providing funding for infrastructure or programme grants and by enacting legislations that encourage donations, such as offering tax credits to local producers who donate surplus food (Tarasuk et al., 2020). However, in line with the neoliberal funding arrangements outlined earlier, the government provides no direct food or core funding to food banks (Tarasuk et al., 2020). While the pandemic saw unprecedented funding from the federal government for charitable food organizations^4^ (Men & Tarasuk, 2021), this support has since stagnated. Food banks are facing increasing financial strain as they struggle with unprecedented demand, driven by inflation and rising living costs, exacerbating concerns over their long-term sustainability (Bogden, 2024; Whitten, 2024). As with the broader non-profit sector, and indeed, all social reproduction labours, food banks find themselves in a liminal space; being both essential to the economy and society by addressing a crucial need and also marginalized within them.

It is clear that food insecurity is a pressing issue in Canada, and that food banks and other food provisioning organizations play an essential role in ensuring that many Canadians do not go hungry. However, charitable food programmes such as food banks have been confronted with significant critique. First, many argue that through food banks, hunger is depoliticized, as the responsibility for the provision of food is shifted from the government to the local community level and to individuals (Long et al., 2020; Riches, 2018). This detracts attention from tackling the root causes and systemic injustices that lead to food insecurity in the first place (May et al., 2019). Second, and relatedly, the normalization of food banks obscures the failure of the state and allows neoliberalism to continue unchallenged (Cloke et al., 2017; Riches, 2018; Strong, 2020). The third line of critique concerns the potential benefits corporations derive from the food bank system. Some point to the food bank system as an avenue through which corporations may avoid costly waste disposal (Spring et al., 2022), while others highlight the incentive structures put in place to encourage corporations to donate surplus food, including tax breaks and tax credits (Lindenbaum, 2016). Finally, the co-optation of food banks by corporations – through the inclusion of business executives on the board of directors or through formalized partnerships – has been identified as a significant issue related to food banking (Azadian et al., 2023; Mendly-Zambo & Raphael, 2019). Fisher (2018) coins this the ‘hunger-industrial complex’, denoting the problematic alliance between anti-hunger organizations and large corporations. This line of critique emphasizes how corporations’ employment practices, anti-union activities, and lobbying for welfare retrenchment directly create and perpetuate food insecurity, and can therefore exert a counterproductive influence on the agendas of food charities (Azadian et al., 2023; Fisher, 2018).

Building from these critiques, we engage particularly with the depoliticization of hunger and the responsibilization of the local community for food provision that allows neoliberalism to continue unchallenged. From a social reproduction perspective, the proliferation of food banks can be considered an iteration of the privatization of social reproduction under neoliberalism as the responsibility for food security is shifted from the state to individuals, families, and communities. We concur with Strong's (2020) argument that food banks are “one vital site ‘taking on the work’ and ‘filling the gaps’ in the emergent landscape of intervention that operates at ground-level to support people in negotiating the impacts of austerity” (p. 211). As a (now decades-long) temporary stop-gap to the subsistence crises elicited by neoliberal austerity, the institutionalization and normalization serves to socially reproduce the same: that is, food banks – and the culture that lauds them – serve to reproduce the political economies that make them necessary. These sites also exemplify the uneven impacts of neoliberal policies, as the material-economic burden of social spending cuts falls disproportionately onto already marginalized individuals and communities. Strong (2020) illustrates this in his study on UK food banks which reveals that the users of food banks are also often the volunteers who staff them. The unpaid care work that occurs in these spaces is deeply shaped by class dynamics, as predominantly low-income individuals both seek assistance and provide it. In this way, Strong (2020) argues that “food banks are centrally involved in the already uneven and exploitative geography of social reproduction, impacting on where, how and by whom the basic subsistence and survival of certain populations is maintained” (p. 211).

While we recognize food banks as products of the uneven processes of neoliberal austerity, and condemn the political-economic ideologies that necessitate them, we are loathe to equate the work – and the workers – of these organizations with the critique of the political economic conditions through which they operate. This is for a few reasons. First, we agree with Regnier-Davies et al.'s (2023) assertion that “the critique of charitable models fails to differentiate between larger-scale corporatized food bank organizations from smaller-scale efforts that are rooted in community and developed in response to community needs” (p. 357), and their insistence that community-based food support initiatives have the potential to “influence and shape more equitable and inclusive food environments and future security” (p. 357). The SOAHAC food bank programme is a pertinent example of this. Richmond and Dokis (2023) highlight how Indigenous food insecurity is shaped by settler colonial policies that disrupt traditional Indigenous foodways, making traditional food in London, Ontario, highly inaccessible. While the food bank programme addresses immediate food needs in culturally safe and dignified ways, its long-term goal is to cultivate Indigenous food sovereignty. It is working to do so by growing traditional foods and medicines, offering cooking classes to teach traditional food preparation, and holding specialized events for cultural knowledge sharing on traditional food systems. This food bank programme represents a community-led approach built by and for Indigenous people that meets immediate needs while reclaiming traditional Indigenous foods and knowledge systems, pushing back against the colonial and neoliberal frameworks that have historically undermined Indigenous communities’ self-sufficiency. However, their reliance on short-term, restrictive grants and unpaid labour – a product of neoliberal funding arrangements – undermines these efforts, illustrating how food banks operate within an against the gendered and racialized constraints of the neoliberal political economy.

Second, we maintain that while workers’ labour – food bank workers, and non-profit and care workers, more generally – is shaped through the multiscalar effects of racial capitalism and neoliberal austerity, this labour is neither necessarily bound by nor reflective of those values; in fact, some may actively seek to challenge and transform them. Swords (2023) details how, through a collaborative action research programme, the Food Bank of Southern Tier (FBST) in New York underwent a ten-year process of organizational restructuring, transforming from a charity model to a rights-based approach by integrating advocacy and political education into their programming and operations. FBST's transformation not only indicates food banks’ potential to shift their discourses and practices beyond charity, but food bank labourer's dedication and will to do so. Swords’ (2023) project was inspired by other research examining the capacity of food banks to evolve into advocacy spaces (Dodd & Nelson, 2020; Galinson, 2018). Others have debated the ability of food banks to mobilize a national or international food justice movement (Spring et al., 2022; Williams et al., 2016). This debate is beyond the scope of this paper; however, what it reveals is the productive tension between food bank workers’ imperatives and visions for their work and the institutional and broader political economic mandates for the same.

Third, we position food banks as not only emblematic of the broader phenomenon wherein non-profit organizations take on critical socially reproductive roles in the absence of adequate state intervention but also as vital ‘shadow care infrastructures’ (Power et al., 2022). As Power et al. (2022) argue, neoliberal welfare retrenchment has necessitated a re-assemblage of caring practices that ensure not merely survival, but also wellbeing and flourishment for those marginalized by austerity measures. We suggest that the diverse landscape of community-led food organizations – including meal service providers, community kitchens, community gardens, and food justice groups – function as shadow care infrastructures, where care extends beyond the immediate alleviation of hunger to encompass broader community support systems. For example, Miewald and McCann (2014), writing on Vancouver, found that community kitchens serve as both sites of material survival and spaces of relational care, where social networks between beneficiaries and staff are formed. In fact, food insecure individuals reported that their choice of where to access free meals was based not only on food availability or proximity, but also on experiences of care, including food quality, sociability, safety, and respectful treatment by staff (Miewald & McCann, 2014).^5^ This reveals the multiplicity of social reproduction work; it is an assortment of intimate labours that reaches beyond the immediate materiality of the labour output to encompass the intangible affective and relational aspects of life-making. Recognizing food organizations as ‘shadow care infrastructures’ in which social reproduction takes place compels a shift in focus – from viewing them as temporary crisis responses to embedded, politically significant spaces where collective care and community-building emerge in response to systemic neglect. This reframing underscores the need to not only acknowledge the agency of non-profit workers and community members but also to hold neoliberal political and economic structures accountable for (seemingly permanently) offloading social reproduction onto precarious, under-resourced spaces.

Fourth and finally, through everyday labour and relations, these community care systems, like other non-profit spaces, may challenge the hegemonic neoliberal ideals of self-sufficiency and individualized narratives of poverty and hunger. Cloke et al. (2017) find that food banks can be spaces of care in which interactions between volunteers and users may foster values like solidarity and generosity, potentially leading to critiques of austerity. Surman et al. (2021) argue the same, but importantly note that such transformations are more common in independent food banks than corporatized ones, echoing Regnier-Davies et al.'s assertion of the importance of recognizing the diversity of the non-profit food provisioning sector. Phillips and Willatt (2020), writing on the U.K., similarly illustrate how the embodied experience of working in community kitchens exposes the political dimensions of food provisioning labour, which can challenge pre-conceived notions about poverty and hunger as a self-inflicted, individual issue and can motivate workers to take political action. These insights echo feminist conceptualizations of social reproduction as a site of transformation.

We approach this transformative potential of food bank workers’ labour through its tension with the conditions in which food banking takes place, and our critique thereof. Food banks (and other non-profits) are sites of care in which essential social reproductive work is being carried out by an invisibilized, feminized, and undervalued workforce, within and at times against the constraints of neoliberal austerity. We highlight the diversity of food bank structures, the evolutionary capacity of food banks, their role in fostering care and community – both of which the neoliberal project seeks to erode – and their potential to cultivate resistance to the ideologies that necessitate them. Most importantly, we argue that while their existence in such abundance represents the continuation of a destructive political-economic ideology, providing food for communities, like other social provisioning activities, is not merely a response to gaps in formal government/market provisioning. Rather, it is agentic life-making labour driven by dictates beyond capital accumulation.

## Conclusion

While food banks were initially conceived as a temporary stop-gap measure in the 1980s and 1990s, in Canada and elsewhere they have become an enduring social institution of the neoliberal era. And, while food banks are positioned as a provider of last resort, contemporary data reveals a drastic upswing in food bank users, reflective of the prevalence of food insecurity across the country (approximately one in five households). The labours of food bank workers (and, indeed, broader food organizations), then, make essential contributions to the social provisioning of Canadian communities and yet they are positioned as a temporary anomaly, an exception to be endured until we return to the “normal” workings of the state and capital. The espoused exceptionality belies the endurance of the care crisis under capitalism, and obscures the essential work being carried out in these spaces. As Fraser (2016) argues, the so-called care crisis, made manifest in skyrocketing levels of food insecurity, is not an exception or temporary blip in the workings of capitalism, but rather is endemic to it. Thus, the imagined temporariness of community care operates alongside its feminized nature and its position straddling the paid/unpaid nexus to devalue and invisibilize this labour. While critiques of the neoliberal genesis and, indeed, corporate operations of many food banks rightly shed light on the austerity politics underpinning the sector, we are concerned with the critiques of these structures erroneously extending to the vibrant community labours within this sector. As such, while critiquing the gendered and racialized capitalist relations that devalue this work, by conceptualizing non-profit labour, in general, and food bank labour, in particular, as a site of social reproduction, we have aimed to reveal the caring relations at this site that resist and extend beyond the imperatives of capital.

Our analysis of food banks provides a case study of the social reproductive labour of non-profits. By proposing that the work of non-profits constitute life-sustaining activities that supplement our subsistence and caring needs, particularly in the case of marginalized communities, we seek to interrogate the multiscalar labours of – and responsibilities for – social reproduction. Whose responsibility is it to socially reproduce a community? Far from a given, the answer to this question is always contested and ever-shifting across place and time. In Canada, there are dominant assumptions around the state's role in ensuring a basic level of social welfare (a level that has never extended to all those living in within Canada's borders and that has, over the last forty years, eroded significantly). Yet the responsibilities for social provisioning are quite clearly being offloaded to the non-profit sector, while the state maintains control over these activities via conditional funding arrangements. This reproduces a gendered and racialized precarity that characterizes the lives and labours of both non-profit workers and the communities whom they serve.

From this analysis, three key implications emerge: First, conceptualizing non-profits as spaces of constrained care under neoliberal austerity highlights the urgent need for policy reforms to, in the short-term, direct funding toward the non-profit and social work institutions that are carrying out essential community reproducing and life sustaining work, and, in the long-term, re-invest in public welfare infrastructure rather than rely on precarious non-profit labour. Second, acknowledging non-profit workers’ agency, not just their exploitation, allows us to identify a diverse workforce that is shaping alternative practices of care, solidarity, and community-building. This points to the importance of supporting collaborative organizing spaces where non-profit workers and community members can co-create services and engage in collective advocacy, resisting neoliberal pressures toward depoliticization and privatized survival. Finally, we encourage further feminist inquiry into the political potential of the non-profit and social care work space – research that elevates these workers’ agency as they navigate the crisis of care, and that calls for a socio-political valuing and funding of programmes that not only play an essential part in the reproduction of communities but also challenge dominant neoliberal ideals through their caring practices.

As the case of food banks illustrates, the tensions between the imperatives of lifemaking and the devaluation of care in our current political economy are not (only) structural, but manifest in our lives and bodies: in hungry people, sick people and people deeply burdened by day-to-day fears of not meeting their needs. Extending an analysis of social reproduction from the home into the community – formal and informal organizations, paid and unpaid labour – reframes non-profit work as the essential work of lifemaking. In doing so, the structural underfunding and precariousness of the sector is made all the more egregious, paradoxically destabilizing the organizations that ensure survival. Nonetheless, the labours of social reproduction are ebullient, and at their core they pose a challenge to the disciplines of capitalism. We have focused on food banks, in particular, because they are an example of social provisioning that has been most critiqued for their state-and-corporate cooptation. And yet, notwithstanding these real limitations, the workers (also often food bank users) – in their day-to-day conversations in the sensuous work of sorting foodstuffs and seasoning stews – articulate an ‘in the meantime’ (Cloke et al., 2017) alternative to the very social conditions through which their institutions were produced.

## Notes

1. Here, we are concerned with what Shields (2014, p. 257) calls the ‘core sector’ of the non-profit world, made up of mostly small, community-based organizations with annual revenues of less than $100,000 and whose mission is to serve a public good in a small geographic region. The core sector excludes organizations such as hospitals, colleges and universities who are closely tied to and regulated by the state (Shields, 2014).

2. It is notable that the terms “social reproduction” and “care” are often used somewhat interchangeably. Here, we use both terms, with social reproduction as our analytic and approach to the labour we are discussing, and “care” as a descriptor of the services provided by many non-profits.

3. During its Keynesian era, the Canadian federal government introduced a myriad of social-security programs and benefits intended to support families and communities, such as the Unemployment Insurance Act (1940), Family Allowances (1945) (which provided monthly installments to families with children), the Canada Pension Plan (1965), and the Medical Care Act, which made healthcare universally accessible. The Canadian Assistance Plan (CAP), implemented in 1966, was particularly significant, as it provided federal funding for provincial social services such as child welfare, family counseling, and daycare (Gill, 2021).

4. This included the $200 million Emergency Food Security Fund, $50 million to support surplus food distribution, and additional funds for food programs in Northern and Indigenous communities (Men & Tarasuk, 2021).

5. By positioning food banks and food organizations as potential community care networks, we do not elide the ‘messy middle ground’ (May & Cloke, 2014) that can emerge in volunteer spaces, including regressive and paternalistic care practices. Rather, we acknowledge that while these spaces are shaped by power dynamics, resource constraints, and structural inequalities, they also remain inherently political, as they navigate and contest the neoliberal retreat of the state, often fostering forms of solidarity and resistance alongside their challenges.
